# Outcomes of the Sustainable Culturally Adapted Nutrition Program (SCAN) and food supplement program to improve engagement with the National Diabetes Prevention Program (NDPP)

**DOI:** 10.3389/fcdhc.2026.1838175

**Published:** 2026-08-24

**Authors:** William B. Perkison, Pierre Fwelo, Natalia I. Heredia, Ella Garza, James J. Yang, Fernanda Velasco-Huerta, Serena A. Rodriguez, Sidra S. Beg, Catherine Pulicken, Patenne D. Mathews, Belinda M. Reininger, Roshanda S. Chenier, Maria E. Fernandez

**Affiliations:** 1 The University of Texas Health Science Center at Houston School of Public Health, Houston, TX, United States; 2 Center for Health Promotion and Prevention Research, The University of Texas Health Science Center at Houston School of Public Health, Houston, TX, United States; 3 Southwest Center for Occupational and Environmental Health, The University of Texas Health Science Center at Houston School of Public Health, Houston, TX, United States; 4 The University of Texas Health Science Center School of Public Health, Brownsville, TX, United States; 5 Department of Biostatistics and Data Science, The University of Texas Health Science Center at Houston School of Public Health, Houston, TX, United States; 6 The University of Texas Health Science Center School of Public Health, Dallas, TX, United States; 7 Institute for Implementation Science, The University of Texas Health Science Center at Houston, Houston, TX, United States

**Keywords:** diabetes, DPP, health education, health promotion, nutrition, nutrition intervention, prediabetes, type II diabetes

## Abstract

**Purpose:**

The National Diabetes Prevention Program (National DPP) is an evidence-based lifestyle program for patients with prediabetes, but low attendance and retention hinder its effectiveness. To improve adherence, we developed the Sustainable Culturally Adapted Nutrition (SCAN) program. SCAN includes 4 group cooking technique lessons and group motivational interviewing counseling to reinforce participants’ ability to adapt and sustain the dietary recommendations discussed in the National DPP. The study aims were to assess 1) the feasibility of incorporating SCAN into the National DPP and 2) health related outcomes for SCAN + National DPP compared to National DPP only.

**Methods:**

We employed a pragmatic quasi-experimental approach for this pilot. 37 National DPP participants were recruited from a Federally Qualified Health Center in Houston, Texas, and were randomized to participate in the intervention or control arms. The primary outcome was National DPP attendance, and the secondary outcomes were weight and body mass index (BMI). Chi-square and Fisher’s exact tests compared baseline characteristics. Linear regression assessed the association between weight and National DPP attendance.

**Results:**

At 12 months, the intention-to-treat and per-protocol analyses found no significant difference in attendance. There was a greater dose-response association between National DPP attendance and average weight loss in the intervention group. Among the SCAN cohort, there were improvements in confidence in assessing personal risk for diabetes and ability to reach healthcare providers.

**Conclusion:**

SCAN’s integration into the National DPP has shown potential in improving attendance and was well received. Larger scale implementation is necessary to understand the long-term impact of SCAN.

## 1 Introduction

Diabetes is a serious public health concern with rising incidence in the United States. In 2019, 28.7 million people in the United States (U.S.) (8.7% of the population) aged 18 years and older self-reported being diagnosed with diabetes, and 96 million people self-reported having pre-diabetes (1). From 2017 to 2020, prediabetes prevalence was high in the general population and in every racial/ethnic group, with the highest prevalence amongst non-Hispanic Black (39.2%), followed by non-Hispanic White (38.7%), non-Hispanic Asian (37.3%), and Hispanic adults (34.5%) (1). Moreover, the general population’s awareness that they have pre-diabetes is also low; according to self-report data, only 19% of the general population that had a diagnosis of prediabetes according to their blood glucose levels were aware that they had prediabetes (1). This level of awareness was distributed evenly among all racial/ethnic groups including non-Hispanic White (17.3%), Hispanic (20.9%), non-Hispanic Black (21.9%), and non-Hispanic Asian adult (30.%) (1). There is a clear need for effective interventions to prevent diabetes in Americans.

The National Diabetes Prevention Program (National DPP) is a 12-month evidence-based lifestyle change program implemented to reduce the incidence of Type 2 Diabetes Mellitus among individuals with prediabetes (2, 3). The program, led by Centers for Disease Control and Prevention (CDC)-trained lifestyle change coaches, teaches participants how to make lifestyle changes to improve their nutrition, become more physically active, and manage their weight and is proven to help participants lose weight in the short term whilst reducing their risk of developing diabetes in the long term (2, 4). Patient-level health outcomes associated with the National DPP were assessed in a study utilizing a nationwide population-based registry dataset. The median weight loss reported for the 4,808 participants who attended at least 17 National DPP sessions over 7–12 months was 6.0%, compared to 1.9% among those who attended 2–16 sessions over 1–6 months of the program (2). Furthermore, a 58% reduction in the incidence of diabetes was demonstrated at the 2.8-year mark and 34% at the 10-year follow-up among National DPP participants who completed the program (3). Thus, evidence suggests that the DPP program is effective and that health outcomes are affected by class attendance and program adherence to program recommendations (2, 4, 5). The benchmark for program adherence is defined as attending a minimum of eight sessions in months 1–6 and attending at least 1 session during months 7-12 (2).

Despite these proven benefits of the National DPP, attendance and adherence are suboptimal in many populations, which reduces the overall efficacy of the programs (2). Program adherence can be partially influenced by the participants’ lack of perceived ability to prepare recipes that align with their cultural identity, and the ability to afford more nutritious food options (6–8). This is concerning, especially among racial and ethnic minority groups who are at higher risk for developing diabetes and traditionally prepare foods differently than those suggested in standard American recipes (6–8). To address these barriers, we have developed the Sustainable Culturally Adapted Nutrition (SCAN) program designed to supplement the existing National DPP (9). The SCAN program includes lessons to improve cooking skills for participants, a motivational interviewing (MI) training program for the cooking instructors, and a food supplement program for participants. Our previous publication summarized preliminary results of the SCAN/National DPP program at 6 months (9). In this paper we describe the individual components of the program in more detail, the study methodology, and report on the 12-month outcomes of the SCAN participants compared with the control group in terms of participant adherence results, changes in biometric measurements, and the results of the satisfaction survey.

## 2 Methods

### 2.1 Design

We conducted a pragmatic quasi-experimental pilot study in an urban Federally Qualified Health Center (FQHC). Enrolled participants were assigned to either an intervention group that included both the National DPP and SCAN programs or a comparison group that included only the National DPP. The UTHealth Committee for the Protection of Human Subjects approved all study procedures (UTH IRB #: HSC-SPH-21-0180).

### 2.2 Sample size

The study was conducted in HOPE Clinic, an FQHC clinic in Houston with over 20 providers that serves over 20,000 patients each year, including ethnic and racial minorities originally from Central and South America, Africa, and Eastern Asia who are primarily uninsured or underinsured (10, 11). HOPE Clinic was selected to take part in this pilot study given its participation in the implementation of referrals to the National DPP (12). The DPP sessions were conducted by First Mile Care. First Mile Care is a private, for-profit organization that conducts the National DPP program in clinic settings nationwide. The target sample size was to recruit 60 participants to fill 4 classes of 15 participants per class of the National DPP.

### 2.3 Intervention

#### 2.3.1 Cooking classes

Cooking class sessions were developed with the Nourish Program, a comprehensive nutrition education program based at the UTHealth School of Public Health. We culturally adapted an existing cooking class curriculum that included recipes and activities (13, 14). The curriculum was developed based on the Social Cognitive Theory and aimed to increase participants’ outcome expectations of the taste of healthy foods, knowledge of healthy eating, self-efficacy for engaging in healthy eating, culinary skills of preparing healthy and flavorful foods, and social support to prepare healthy foods (14, 15). Guided by Intervention Mapping, a systematic process for adapting, developing, implementing, and evaluating theory- and evidence-based interventions, the team identified both surface-level and deep-level adaptations needed to ensure curriculum materials and activities were culturally appropriate (16). Surface-level adaptations to cooking classes included tailoring of educational and promotional material such as recipes, pamphlets, and images to include content that would resonate with the participants’ cultural background and offering classes in Spanish and English. Deep-level adaptations included the integration of MI by the SCAN coaches throughout the course delivery to meet participants’ needs.

### 2.4 Training cooking coaches in SCAN and motivational interviewing

SCAN cooking class coaches received training in both program delivery and MI, a communication method to engage participants in meaningful conversations that aim to inspire behavior change (17). Specifically, a Nourish registered dietitian trained SCAN cooking class coaches through lectures, hands-on practice, and case study discussions to increase SCAN coaches’ knowledge in nutrition communication and culinary medicine (18). The training was individualized to increase the SCAN coaches’ ability to make meaningful cultural adaptions while simultaneously delivering the cooking lessons. SCAN coaches needed to be flexible and customize the course content to meet the needs of participants in real-time. Conducting systematic modification using this strategy ensured that the lessons resonated with the participants’ language, culture, context, cultural patterns, meanings, and values (19). SCAN coaches participated in 14 hours of motivational interviewing training and consultation (May 2021 – March 2022) that included: 1) motivational interviewing history and theory; 2) the principal tenets of behavior change counseling; and 3) demonstration and practice of motivational interviewing techniques (e.g., asking open questions, affirming participants’ strengths, and using reflective listening to strengthen participants’ self-efficacy to develop healthy behaviors).

Coaches also participated in counseling demonstrations to learn how to effectively counsel SCAN participants during the cooking sessions. The coaches used open-ended questions to elicit change talk, such as “How might you make this change?” and “So what are your next steps?” Research has indicated that motivational interviewing has been found to be effective in strengthening behavior change among patients, while also promoting adherence (17).

### 2.5 Recruitment and enrollment

Recruitment took place between July and September of 2021. National DPP participants referred by the FQHC were contacted via telephone to participate in SCAN. National DPP eligibility was determined through 2 sets of criteria; patients needed to meet all of the initial eligibility criteria as well as satisfy an additional requirement outlined below (9). Patients needed to satisfy all the following eligibility criteria: 1) be at least 18 years of age, 2) have a body mass index (BMI) greater than 25, 3) not have a prior diagnosis of Type I or Type II Diabetes, and 4) not be pregnant. Additionally, an eligible patient must satisfy at least 1 of the following requirements: 1) obtain a blood test result in the prediabetes range (Hemoglobin A1C (HgbA1C): 5.7–6.4%), 2) have a previous diagnosis of gestational diabetes, or 3) attain a Prediabetes Risk Test score greater than 5.

Eligible participants were recruited through EHR-based screening and provider referral processes. Participants selected either English- or Spanish-language classes based on their preference. After all classes reached the target enrollment of 15 participants, classes were randomized to either the intervention (SCAN + DPP) or control (DPP only) condition.

Randomization was stratified by DPP start date and language preference to ensure balanced representation of these characteristics across study arms. Stratification was used to reduce potential confounding related to differences in program timing or language-specific delivery. Because all classes were enrolled prior to randomization and the first classes did not begin until all classes had been assigned, the potential influence of start date on participant outcomes was considered minimal.

Questions regarding race and ethnicity were listed as fixed categories. Personnel who enrolled and those who assigned participants to the interventions had access to the random allocation sequence. This pilot trial was not blinded. An onboarding packet was mailed to the intervention participants, which included information about the food supplement program, a food supplement identification card, a list of food pick-up locations, a Nourish recipe book, and written consent forms.

### 2.6 Program delivery

#### 2.6.1 Food supplement program

Both intervention and control arms participants were enrolled in the food supplement program run by the Houston Food Bank. The Houston Food Bank currently distributes over $200 million of food each year to over 800,000 recipients with food insecurity via 1600 associated food distribution sites (20). The Houston Food bank also currently partners with approximately 30 different health care outpatient clinic systems in the Houston area. Providers in these healthcare systems give a “food prescription” to their patients, which is reimbursed at 1 of the local distribution centers for 30 pounds of fresh fruits and vegetables, twice a month (21). In our study, enrolled participants received a food supplement ID card, which they used to obtain food vouchers, twice per month, for the duration of the National DPP at 1 of several different local distribution centers. Because we enrolled participants directly into our program and not through the clinic providers, we did not include the provider’s “prescription”, so in this paper, our version of the program is termed a *food supplement* program. A food supplement program was included in the study to lower barriers for participants’ access to the healthy foods that they were learning about in both National DPP and SCAN programs and to reinforce the participant’s willingness to make changes (22–25).

#### 2.6.2 Cooking class instruction

SCAN coaches delivered 4 1-hour cooking classes virtually to participants in the intervention group. All SCAN sessions were held during the first 6 months of the National DPP program. Participants were given session information at study enrollment, including the link to connect to the virtual cooking classes. Incentives (i.e., cooking class recipe ingredients and cooking tools) were delivered prior to each class to promote participation and eliminate food availability barriers. Participants were encouraged to follow along with the cooking sessions using the recipe ingredients and cooking tools provided by the SCAN program and delivered to their homes the day of the cooking class. A summary of each class and its components are presented in **Table 1**.

**Table 1:** Sustainable culturally adapted nutrition program (SCAN) culinary classes summary.

SCAN session	Purpose	Recipe	Takeaways
Knife skills	Participants will learn and practice safe and effective knife skills to cut a variety of vegetables into even sizes.	Texas Caviar with dressing	Utilize the entire vegetable to reduce waste
Roasting	Participants will learn the step-by-step process of how to prepare and roast flavorful vegetables.	Tajin Spiced Roasted Carrots	Don’t peel the vegetables
Sautéing	Participants will learn the step-by-step process of how to safely prepare and sauté flavorful vegetables.	Tajin and Oregano Sautéed Winter Vegetables	Recipes and ingredients can be customized to patient preference
Microwaving	Participants will learn how to follow the step-by-step directions to cook flavorful vegetables in the microwave.	Garlic and herb butter broccoli	Cut vegetables uniform size for even cooking

### 2.7 Measures

At the completion of the SCAN intervention (6 months) and the National DPP intervention (12 months), participants in both study arms completed a telephone-administered survey conducted by trained research staff. The survey instrument was adapted from the Nourish Culinary Medicine evaluation survey, which was originally developed to assess participant experiences with culinary medicine programming (14).

The survey evaluated several domains, including participant satisfaction, perceived usefulness of the program, comfort with virtual participation, self-efficacy related to healthy eating and culinary medicine skills, and knowledge gained regarding lifestyle behaviors.

Responses were measured using a 5-point Likert scale, with higher scores indicating greater agreement, confidence, satisfaction, or perceived benefit. Example items included: “The course was helpful to me” (1 = strongly disagree to 5 = strongly agree), “How confident do you feel using Zoom?” (1 = not confident at all to 5 = very confident), and “How confident do you feel about shopping for healthy foods?” (1 = not confident at all to 5 = very confident). Participants also had the opportunity to provide open-ended qualitative feedback regarding their experiences with the program.

Survey responses were collected and managed using REDCap. The final instrument consisted of 23 items. 12 items assessed participant satisfaction with the SCAN intervention, the National DPP, the DPP lifestyle coach, the food supplement program, and overall program progress. 8 items measured self-efficacy related to diabetes risk assessment, healthy food shopping, healthy meal preparation, culturally relevant healthy cooking practices, and program participation. 3 items evaluated knowledge gained regarding healthy eating, physical activity, and stress management.

### 2.8 Analysis

Our goal for this study was to pilot the incorporation of SCAN into the National DPP and to measure its impact on National DPP attendance. The primary outcome of interest was comparison of attendance rates between the intervention and control group, measured at the end of the National DPP program (12 months). Secondary measures include anthropometric (weight and BMI) and biometrics (HgbA1C, low-density lipoprotein cholesterol (LDL-C), high-density lipoprotein cholesterol (HDL-C), and triglycerides) outcomes. We abstracted anthropometric and biometric measurements from the HOPE Clinic EHR at baseline; these measures were not conducted at follow-up due to attrition within the cohort. In addition, weight was self-reported by patients to the National DPP provider during each DPP class. Lastly, we administered survey questions to gain a better understanding of the patients’ National DPP/SCAN satisfaction, knowledge, and self-efficacy.

We first performed descriptive analyses to describe participants’ sociodemographic characteristics (age, race, sex, ethnicity, education, and language), anthropometric measures at baseline (weight, blood pressure, BMI, HgbA1C, HDL-C, and LDL-C), and Prediabetes Risk Test [yes/no]. The data were summarized using mean and standard deviation for continuous variables and frequency for discrete variables. We compared the differences between the intervention and control group using a two-sample t-test for continuous variables and a Chi-square or Fisher’s exact test for discrete data.

We used linear regression models to test the association between National DPP attendance and SCAN study groups (intention-to-treat and per protocol). For the intention-to-treat analysis, we compared the National DPP attendance of patients in the SCAN plus National DPP intervention group to those in the comparison group (National DPP only) group. In the per protocol analysis, we compared the National DPP attendance of patients who attended at least 1 SCAN cooking class to those who did not. In addition, a linear mixed model was performed to test the association between National DPP attendance and weight loss over time to account for the correlation of repeated measures within each subject. We graphed the participants’ average percent weight change from baseline over time, stratified by study group.

## 3 Results

Of the 61 participants enrolled in SCAN, 37 attended at least 1 National DPP class. The other 24 participants were excluded from the analysis due to missing baseline data. As detailed elsewhere, participants in the intervention group were younger (46.6 ± 12.20 vs. 50.47 ± 9.06; p*> 0.05*), more likely to be female (72.70% vs. 60%; *p> 0.05*), and Hispanic (73.7% vs. 33.3 %; *p> 0.05*) compared to those in the control group (**Table 2**) (9). Chi-square and Fisher’s exact tests comparing the sociodemographic characteristics and baseline anthropometric measures between the control and intervention groups revealed no statistically significant associations. In the per-protocol comparison, those who attended at least 1 SCAN cooking class were younger (44.83 ± 13.64 vs. 49.89 ± 12.2; p*> 0.05*), more likely to be female (75% vs. 64%; *p> 0.05*), and Hispanic (58.33% vs. 52.00%; *p> 0.05*) compared to those in the control group.

**Table 2:** Baseline characteristics of the DPP participants enrolled in the socially culturally adapted nutrition (SCAN) program (N = 37).

	Intention-to-treat			Per-protocol		
Participant baseline characteristics	Control (n=15) N (%)	Intervention (n=22) N (%)	p-value^1^	Attended 1+ cooking class (n=12) N (%)	Attended 0 cooking class (n=25) N (%)	p-value^2^
Age [mean(std)]	50.47 (9.06)	46.6 (12.20)	0.308	44.83 (13.64)	49.80(12.20)	0.205
Race			0.082			0.049
African American or Black (std)	6 (40.00%)	5 (22.73%)		4 (33.33%)	13 (52.00%)	
White (std)	4 (26.67%)	15 (68.18%)		8 (66.67%)	7 (28.00%)	
Other/Not reported	5 (33.33%)	2 (9.09%)		0 (00.00%)	5 (20.00%)	
Sex			0.650			0.769
Male	6 (40.00%)	6 (27.30%)		3 (25.00%)	9 (36.00%)	
Female	9 (60.00%)	16 (72.70%)		9 (75.00%)	16 (64.00%)	
Ethnicity			0.077			0.755
Hispanic	4 (26.67%)	15 (63.63%)		7 (58.33%)	13 (52.00%)	
Not Hispanic	8 (0.53 %)	5 (22.73%)		4 (33.33 %)	9 (36.00%)	
Other/Not reported	3 (40%)	2 (13.64%)		1 (8.33%)	3 (12.00%)	
Education			0.432			0.386
Less than grade 12	0 (0%)	1 (4.55%)		1 (8.30%)	0 (0.00%)	
Grade 12 or GED	1 (6.67%)	2 (9.09%)		2 (16.70%)	1 (4.00%)	
Some college or technical school	3 (20.00%)	1 (4.55%)		1 (8.30%)	3 (12.00%)	
College or technical school graduate	1 (6.67%)	2 (9.09%)		1 (8.30%)	2 (8.00%)	
Not reported	10 (66.67%)	16 (72.73%)		7 (58.30%)	19 (76.00%)	
Language			0.280			0.280
English	9 (60.00%)	8 (36.36%)		6 (50.00%)	11 (44.00%)	
Spanish	6 (40.00%)	14 (63.64%)		6 (50.00%)	14 (56.00%)	
Risk Test			1.000			0.634
Yes	4 (26.70)	5 (22.70)		4 (33.33 %)	5 (20.00%)	
No	11 (73.30)	17 (77.30)		8 (66.67%)	20 (80.00%)	
Weight [mean(std)]	211.60 (42.37)	199 (65.50)	0.541	194.08 (56.99)	211.70 (54.73)	0.065
BMI [mean(std)]	33.92 (6.56)	34.12 (10.01)	0.401	32.38 (8.36)	34.74 (9.17)	0.473
HgbA1C [mean(std)]	5.98 (0.30)	5.90 (0.21)	0.406	5.83 (0.16)	5.97 (0.25)	0.104
Systolic Blood pressure [mean(std)]	129.75 (20.32)	126.40 (13.93)	0.584	123.25 (19.91)	130.30 (17.93)	0.245
Diastolic Blood pressure [mean(std)]	79.50 (6.10)	80.25 (7.06)	0.762	78.75 (6.30)	80.70 (6.86)	0.429
Mean Arterial pressure [mean(std)]	96.25 (10.56)	95.63 (8.77)	0.860	93.58 (7.99)	97.23 (6.86)	0.291
HDL [mean(std)]	54.75 (21.09)	53.55 (15.18)	0.853	54.00 (13.06)	54.00(19.75)	1.000
LDL [mean(std)]	109.25 (30.37)	112.75 (36.14)	0.781	103.92 (33.88)	134.00 (33.88)	0.065

Std, standard deviation, 1control vs intervention, 2control plus intervention group attending 0 SCAN classes vs intervention (attending at least 1 SCAN class).

The intention-to-treat analysis revealed no statistically significant difference in the number of National DPP classes attended between the intervention (7.64 ± 5.49) and comparison group (7.80 ± 7.06) (**Table 3**). The per-protocol analysis showed that National DPP attendance was higher among participants who attended at least 1 SCAN cooking class (9.67 ± 7.69) compared to those in the control group (6.88 ± 5.49), although this difference was not statistically significant (**Table 3**). Although not significant, attendance to each additional National DPP session reduced participants’ average weight by 1.3% (**Table 4**). The study also revealed a greater dose-response association between the number of National DPP sessions attended and participants’ average weight loss among participants in the intervention group compared to those in the control group. Because over 80% of the participants did not have twelve-month biometric data for HgbA1C, HDL and LDL cholesterol, the outcomes analysis was not reported.

**Table 3:** DPP attendance at the 12 months mark of the DPP participants enrolled in the socially culturally adapted nutrition (SCAN) (N = 37).

	Intention-to-treat			Per-protocol		
Participant baseline characteristics	Control	Intervention		Attended 0 cooking class	Attended >1 cooking class	
	(n=15) N (%)	(n=22) N (%)	p-value^1^	N (%)	N (%)	p-value^2^
DPP Classes Attended [mean(std)]	7.80 (7.06)	7.64 (4.60)	0.875	6.88 (5.49)	9.67 (7.69)	0.197
SCAN Cooking Classes	n/a	1.82 (1.79)	n/a	0.00 (0.00)	3.33 (0.81)	< 0.001

Std, standard deviation, 1control vs intervention, 2control plus intervention group attending 0 SCAN classes vs intervention (attending at least 1 SCAN class).

**Table 4:** Longitudinal association log (Weight) on diabetes prevention program (DPP) sessions attended and time in days.

	Model 5 (β estimate)	p-value
Time (in days)	-0.0002	< 0.0001
DPP Session Attended	-0.013	0.08

Model 5: Model 5: log (Weight)= DPP + time.

34 SCAN participants completed the 6-month satisfaction survey, and 13 of these participants completed both the 6-month and 12-month surveys. 5 of the participants who completed both surveys completed them in English, and 8 of the participants who completed both surveys completed them in Spanish. The satisfaction survey revealed that participants were satisfied with the SCAN program (i.e., National DPP and cooking classes), emphasizing positive experiences in learning healthy habits, interactive courses, and group support. 100% of respondents who completed both surveys indicated that they would recommend the program to others, that the SCAN program and National DPP classes kept them involved and interested, and that they will be able to utilize what they learned in the program. At the 12-month vs. 6-month assessments, there was an increase in participants who felt confident or very confident in their ability to assess their personal risk for diabetes (84.62% vs. 77%), to reach their healthcare providers (76.92% vs. 62%), and to cook healthy foods (100% vs. 92%). Conversely, at 12-months vs 6- month assessments, there was a decrease in participants who felt confident or very confident in their ability to shop for healthy foods (69.23% vs. 92%) and to cook foods from their culture in a healthy way (76.92% vs. 92%). There was no change in confidence about skills and knowledge of cooking foods in a healthier way.

While participants in both groups reported gains in knowledge related to lifestyle modification, individuals who attended at least 1 SCAN cooking class and completed the post-intervention survey specifically highlighted the acquisition of practical cooking skills that supported the adoption of healthier dietary behaviors. Overall, participant feedback regarding the SCAN program was highly positive. When asked, “What did you like best about the course?” 1 participant summarized the experience as follows: “I liked all the information they gave about diabetes, the education, the knowledge, the motivation they gave us to move forward, to change diets for the personal good, the sympathy, the responsibility one should have for oneself, the information about how one should live with that disease, and the inspiration they give one to move forward, the reasons to educate oneself.”

Post-intervention survey responses also identified several barriers to participation in both the National DPP and SCAN cooking classes. The most frequently reported challenges included work schedule conflicts and personal responsibilities, such as childcare obligations. Participants who did not attend any SCAN cooking sessions reported greater difficulty maintaining regular participation than those who attended at least 1 session. At the 12-month assessment, 30.8% of respondents identified work schedules and program timing as the primary barriers to participation. Financial constraints were also reported as obstacles to obtaining healthy foods needed for weight management. Although all participants received access to a food supplement program card, fewer than 15% used the benefit, citing the limited quantity of food provided and scheduling difficulties at the food bank. These findings highlight important logistical and structural barriers that may affect program engagement and suggest opportunities for improving future implementation efforts.

## 4 Discussion

This study has demonstrated the feasibility of integrating SCAN into the curriculum of a National DPP. Although there was no statistically significant difference in attendance between intervention and control groups, there was a positive trend in attendance among those participating in SCAN and there were several positive outcomes that showed SCAN’s potential for improving the success rates of the National DPP. First, the participants’ feedback on SCAN was uniformly positive and they felt SCAN enhanced their experience of the National DPP.

Through use of MI strategies, participants were actively engaged in cooking and conversation during the lessons, and it provided an opportunity for them to learn from each other. These conversations addressed the barriers inherent with adopting a new diet on 2 levels. The lessons themselves addressed the technical aspects of the cooking skills needed to properly prepare healthy fruit and vegetable dishes. Additionally, the MI skills acquired by the instructor allowed them to affirm and reinforce the importance of the types of foods rooted in their cultural upbringing and channel their motivation to creatively come up with new recipes that effectively transform important, traditional foods with low calorie, affordable alternatives. Second, those members of the intervention group who made it to at least 1 of the SCAN sessions demonstrated a nonsignificant increase in the number of National DPP sessions attended. While selection bias may partially account for the improved numbers in this group, it also suggests that when participants are successfully able to attend these classes, they will achieve higher National DPP attendance rates and eventually lower rates of developing diabetes.

### 4.1 Limitations

Although this pilot study provided evidence that the SCAN program can make a positive impact on the short-term outcomes of the National DPP, there were some limitations. The low number of participants enrolled in the study resulted in low statistical power to detect significant differences in adherence to the National DPP (outcome of interest). In our recruitment process, we did not objectively test a participant’s proficiency in language prior to enrolling them in either a Spanish or English-speaking class and regarding gender, did not specify if gender was assigned at birth and gender identity was not collected. The lack of allocation concealment during participant enrollment may have resulted in preferential assignment of individuals with greater enthusiasm for the SCAN program to the intervention group, potentially introducing selection bias.

We were unable to obtain baseline data on those 24 individuals who did not attend either National DPP or SCAN session, which could have affected our intention-to-treat analysis. Most of our study was also conducted during the COVID-19 pandemic, which affected many aspects of the study design and could affect our participation and outcome rates. For instance, we were only able to deliver both the SCAN and the National DPP program virtually at a time when many participants had never participated in a virtual call before. Many of our participants only had a smart phone to participate with which limited their ability to simultaneously visualize the class instructors while also preparing their recipes at home. These factors may have affected retention rates in the program as well as the outcomes of the survey results. In our study design, the National DPP program was conducted independently of the SCAN program by First Mile, an organization independent of our research group. This model may be very applicable for some health care organizations who need to partner with outside entities to cover all the various components of the program. However, these outcomes may be different from organizations that incorporate both National DPP and SCAN into a single program. Having a separate entity present the National DPP limited our ability to completely synergize our SCAN program with the contents of the prediabetes programming which may have decreased the impact of our intervention. We also could not control the types of food or how food was distributed in the food supplement program, limiting our ability to reinforce types of food discussed in the program with what was available to participants. Having a food supplement program which emphasizes the foods in the recipes discussed in the program would potentially greatly increase the impact of the intervention. Additionally, we were limited by time, budget, and staffing in being able to include ingredients that were important, culturally, to individual participants in the class. We had similar limitations in being able to offer the classes at a variety of different times and formats to best accommodate participants’ schedules to maximize attendance. We were also not able to follow up with participants beyond the initial 12 months of the program to measure longer term outcomes.

While there were limitations in our ability to control all the variables, these are common issues in community-based programs, and our study may provide useful methods for overcoming these barriers. We will also plan to control for these factors and collect additional data in future studies.

### 4.2 Strengths

The prospective pragmatic study design and clinic environment provided the SCAN program an excellent setting to pilot and further refine its development. This study’s clinic population provided a culturally diverse population that that SCAN program was designed to address. During the program participants were able incorporate their own unique cultural backgrounds into the context of the lesson to provide interactive sessions. We were also fortunate to work with HOPE clinic, a partner that was committed to learning the SCAN material and actively incorporating MI into their presentation. Moreover, the clinic system had its own chef and cooking facilities with dedicated counselors. For health care systems contemplating enacting the National DPP-SCAN program, it is important to have the staffing capacity and facilities available to conduct the program to ensure its success. Our study was also able to follow participants out to 12 months after the program for self-reported weight and attendance data.

### 4.3 Future studies

Future studies are warranted to further refine SCAN. From this initial pilot, several important adaptations to SCAN will be included in subsequent studies. Regarding study design, a larger study over a longer period, such as 6 to 12 months after the completion of the DPP, will be able to better evaluate the effectiveness of SCAN. Evaluating at 6 and 12 months after the completion of the National DPP program with a larger sample size will allow us to better ascertain if improvements in biometric measurements are sustainable and perhaps independent of sessions attended. Also, to improve attendance, future iterations of SCAN will look at how the 4 classes are best spaced out through the 12-month National DPP to optimize attendance rates. The amount of MI training and practice that SCAN teachers receive will also be increased and measured objectively using scoring methods developed for the purpose (26). In addition, future design of the program will incorporate structured group-sharing sessions, as well as an objective feedback workflow to integrate participants’ input and preferences into session components, thereby enhancing cultural appropriateness. We also plan to offer SCAN in a hybrid format at multiple times to maximize accessibility and accommodate participants’ scheduling needs.

Lastly, we relied on provider-ordered clinical lab tests as part of routine primary care visits, but to establish effectiveness of SCAN, it may be necessary to obtain our own project measurements to achieve higher follow up rates at regular intervals.

### 4.4 Conclusions

SCAN shows promise as an effective strategy to increase attendance and adherence to the National DPP, hopefully resulting in enhanced protection against the development of diabetes and its subsequent devastating health complications. SCAN may also help address many barriers experienced by DPP participants.

## Data Availability

The raw data supporting the conclusions of this article will be made available by the authors, without undue reservation.
